# Effectiveness and safety of Ayurvedic intervention in essential hypertension: a systematic review with meta-analysis

**DOI:** 10.3389/fphar.2025.1695614

**Published:** 2025-12-19

**Authors:** Saylee Deshmukh, Kuldeep Choudhary, Azeem Ahmad, Govind Reddy, B. C. S. Rao, Narayanam Srikanth, Rabinarayan Acharya

**Affiliations:** 1 CCRAS-RRAP Central Ayurveda Research Institute, Mumbai, India; 2 Central Council for Research in Ayurvedic Sciences, New Delhi, India

**Keywords:** essential hypertension, ayurveda, systematic review, systolic blood pressure, diastolic blood pressure, herbal medicine, panchakarma

## Abstract

**Background:**

Essential hypertension (EH) is a major contributor to cardiovascular morbidity and mortality that has become a public health challenge owing to poor control and adherence. Many clinical trials have exhibited the effectiveness of Ayurvedic formulations and procedures in the management of EH. In this systematic review and meta-analysis, we present an evaluation of the effectiveness and safety of Ayurvedic interventions compared to conventional therapies for EH.

**Method:**

The systematic review and meta-analysis presented herein adheres to the PRISMA guidelines. Accordingly, we conducted a literature search on PubMed, Cochrane Library, Directory of Open Access Journals, Google Scholar, AYUSH Research Portal, and Ayurveda Research Database for published works up to May 2025. The studies included randomized and non-randomized controlled trials, observational, and pre–post studies of Ayurvedic interventions for EH. The primary outcomes evaluated were systolic and diastolic blood pressure (BP) changes. The risk of bias of ten randomized controlled trials (RCTs) included in the meta-analysis was assessed using the Cochrane guidelines. The meta-analysis was performed in two groups, namely, Ayurveda vs. placebo and Ayurveda vs. standard antihypertensives, using RevMan 5.4.

**Result:**

A total of 44 studies was included in the systematic review, and ten RCTs involving 524 participants were included in the meta-analysis. The interventions included single herbs, polyherbal/herbomineral formulations, and *panchakarma* therapies. Compared to placebo (n = 118), Ayurveda showed a non-significant reduction in systolic BP (mean difference (MD) = −2.63 mmHg; 95% confidence interval (CI): −6.04 to 0.79; *p* = 0.13) and diastolic BP (MD = −2.67 mmHg; 95% CI: −7.44 to 2.09; *p* = 0.27). Compared to standard antihypertensives (n = 396), the reductions in systolic BP (MD = −0.22 mmHg; 95% CI: −0.82 to 0.38; *p* = 0.47) and diastolic BP (MD = −0.66 mmHg; 95% CI: −1.67 to 0.35; *p* = 0.20) values were non-significant. High heterogeneity (I^2^ > 90%) was observed during analysis.

**Conclusion:**

This systematic review shows that although Ayurvedic interventions do not achieve significant BP reductions versus conventional treatments, they may provide clinical benefits with good safety. The main limitations of the present review are the heterogeneity and methodological differences among the different studies. Hence, high-quality multicenter RCTs with standardized interventions are needed to assess the overall effectiveness of these therapies.

**Systemetic Review Registration:**

https://www.crd.york.ac.uk/PROSPERO/view/CRD42019123886, Identifier PROSPERO.

## 1 Introduction

Hypertension is a significant public health concern owing to its associations with coronary heart disease, stroke, and chronic heart disease (Arima et al., 2011). Accordingly, it has emerged as the leading cause of mortality globally and ranks as the third leading cause of adult disabilities (Bostrom et al., 2016). Hypertension remains inadequately managed owing to insufficient awareness, inadequate primary care, and poor adherence (World Health Organization, 2025). Essential/primary/idiopathic hypertension is a multifactorial disorder characterized by sustained chronic elevation of the arterial blood pressure (BP), which comprises almost 95% of all hypertensive cases (Messerli et al., 2007).

The overall prevalence of essential hypertension (EH) in India is 29.8% (Anchala et al., 2014); estimates indicate that approximately 17.6% of these individuals reside in India, suggesting a likely significant increase in the burden of cardiovascular diseases in the near future (Ramakrishnan et al., 2019).

The main factors contributing to elevated BP include obesity, high salt intake, stress, low potassium intake, low calcium intake, excessive alcohol consumption, aging, and insulin resistance (Carretero and Oparil, 2000). Hypertension is often referred to as the “silent killer” in modern society and has significant health implications.

The primary therapeutic approach for managing EH involves regular administration of oral antihypertensive medications. Nevertheless, patient adherence is often suboptimal owing to the high costs and varying degrees of adverse effects associated with long-term medication use, such as hypokalemia, bronchospasm, and angioedema (Hypertension Writing Group of Chinese, 2016). Consequently, there is a need for effective and safe therapeutic alternatives.

Ayurveda is a traditional medical system that has been widely practiced in the Indian subcontinent; it is primarily aimed at holistic management of health and diseases and has historically emphasized health preservation in ancient India. It encompasses a holistic medical framework aimed at regulating the body’s homeostatic mechanisms through various procedures, techniques, medicines, regimens, and dietary measures to achieve specific effects (Sridharan et al., 2011).

There are no direct references to EH in existing Ayurvedic texts. However, based on symptomatology, EH can be correlated with various abnormal behavioral traits described in these texts under the features of *vatarakta* (SAT: ED-8), *raktagata vata* (SAT: AAE-8), and *raktavritta vata* (SAT: AAD-3.1) (Kamble et al., 2018). Based on an understanding of Ayurvedic pathophysiology, it may be said that EH occurs due to vitiation of *vata* (i.e., body factor responsible for movement and cognition; SAT: B.384) and *rakta* (i.e., blood tissues; SAT-B.429), which cause vasoconstriction through impaired hormonal actions and lead to increased peripheral resistance (Menon and Shukla, 2018).

In recent years, various clinical trials published on Ayurveda have shown its effectiveness in reducing BP; there are also some systematic reviews on the effectiveness of certain procedures like *shirodhara* (Khapre et al., 2025). However, to date, there have been no comprehensive systematic reviews on assessing the quality of these published studies, the strengths of their clinical effects, and summarizing the overall evidence for the effectiveness and safety of Ayurvedic interventions in EH. Given these as the main goals, we conducted a systematic review and meta-analysis of the existing studies.

## 2 Methods

This study was registered on PROSPERO with the registration number CRD42019123886, and the protocols used in this review are in accordance with a previous publication (Deshmukh et al., 2019).

This systematic review was conducted according to the guidelines of the Preferred Reporting Items for Systematic Reviews and Meta-analyses (PRISMA) to ensure sufficient quality of evidence (PRISMA, 2020).

### 2.1 Eligibility criteria

#### 2.1.1 Study

We included randomized and non-randomized control trials (RCTs and NRCTs), pre–post single-group designs, and observational studies with Ayurveda as an intervention for essential hypertensive patients aged 18 years and above with no other comorbidities. The language of the published studies was restricted to English.

#### 2.1.2 Participants

Adults with EH were included in this study. According to the JNC VII guidelines on the definition of hypertension, individuals are categorized as hypertensive when their systolic blood pressure (SBP) is equal to or greater than 140 mmHg or their diastolic blood pressure (DBP) is equal to or greater than 90 mmHg (Chobanian et al., 2003).

#### 2.1.3 Intervention

The term “Ayurvedic intervention” as used herein includes treatments or practices based on the fundamental principles of Ayurveda through the usage of internal or external medications, *Panchakarma* (purificatory procedures)*,* administration of single or polyherbal or herbomineral formulations as mentioned in the Ayurvedic texts or Ayurvedic Pharmacopeia of India (API) and administered in the classical forms (such as powder, decoction, and tablet) or as their extracts (Choudhary et al., 2023).

#### 2.1.4 Control/comparator

The control/comparator group comprised placebo or standard antihypertensive treatments or Ayurveda treatments different from those used in the intervention arm.

#### 2.1.5 Outcome measures

The primary outcome measures were improvements in the SBP and DBP from baseline to last follow-up.

#### 2.1.6 Exclusion criteria

Ayurvedic review articles on hypertension, studies on secondary hypertension with other comorbidities, studies containing Ayurvedic interventions as add-ons to standard antihypertensives or other AYUSH interventions like Yoga, Homeopathy, Siddha, and Unani, as well as other systems of medicines practiced globally (e.g., Chinese medicine) but not based on the fundamental principles of Ayurveda were excluded from this survey; moreover, we excluded animal studies and duplicate publications reporting the same groups of participants.

### 2.2 Search strategy

We included databases like PubMed, the Cochrane Library, Directory of Open Access Journals (DOAJ), Google Scholar, AYUSH research portal, and Ayurveda Research Database (ARD) in the search from their inception up to May 2025 for the systematic review and meta-analysis. The search strategy was centered around search terms describing Ayurvedic interventions and EH, namely, ((primary hypertension) OR (essential hypertension)) AND ((ayurveda) OR (herbal medicine) OR (plant)). The strategy was modified for each database as necessary, and the list of search terms used is provided in Supplementary Appendix 1.

### 2.3 Study selection and data extraction

After eliminating duplicate records, titles, and abstracts from the database search results, we screened the remaining search items through a comprehensive examination of potentially relevant abstracts in accordance with the established inclusion and exclusion criteria. The screening and full-text reviews were conducted independently and in duplicate by two researchers (SD and KC), and the decisions were finalized through consensus among all authors.

### 2.4 Risk of bias

The risk of bias was assessed according to the evaluation criteria specified by the Cochrane Handbook for Systematic Reviews of Interventions (Higgins and Green, 2009); accordingly, we examined the random sequence generation, allocation concealment, incomplete outcome data, blinding (participants, personnel, and outcome assessor), selective reporting, and other biases. Two of the authors (SD and KC) independently assessed the included studies and judged each of the domains as having low, high, or unclear risk of bias. The findings were then summarized via the “risk of bias summary” and “risk of bias graph.”

### 2.5 Statistical analysis

The data were analyzed using RevMan 5.4 software provided by the Cochrane Collaboration. For continuous variables, the standardized mean difference (MD) and 95% confidence interval (CI) were employed for the statistical analyses. The heterogeneity of each outcome was assessed using the Chi-squared test and I^2^ statistics. In instances where no significant heterogeneity could be identified (*p* > 0.1 and I^2^ < 50%), a fixed-effects model was utilized for the meta-analysis. Conversely, when heterogeneity was detected (*p* < 0.1 and I^2^ ≥ 50%), a random-effects model was applied along with cautious interpretations.

## 3 Results

### 3.1 Literature search

**FIGURE 1:**
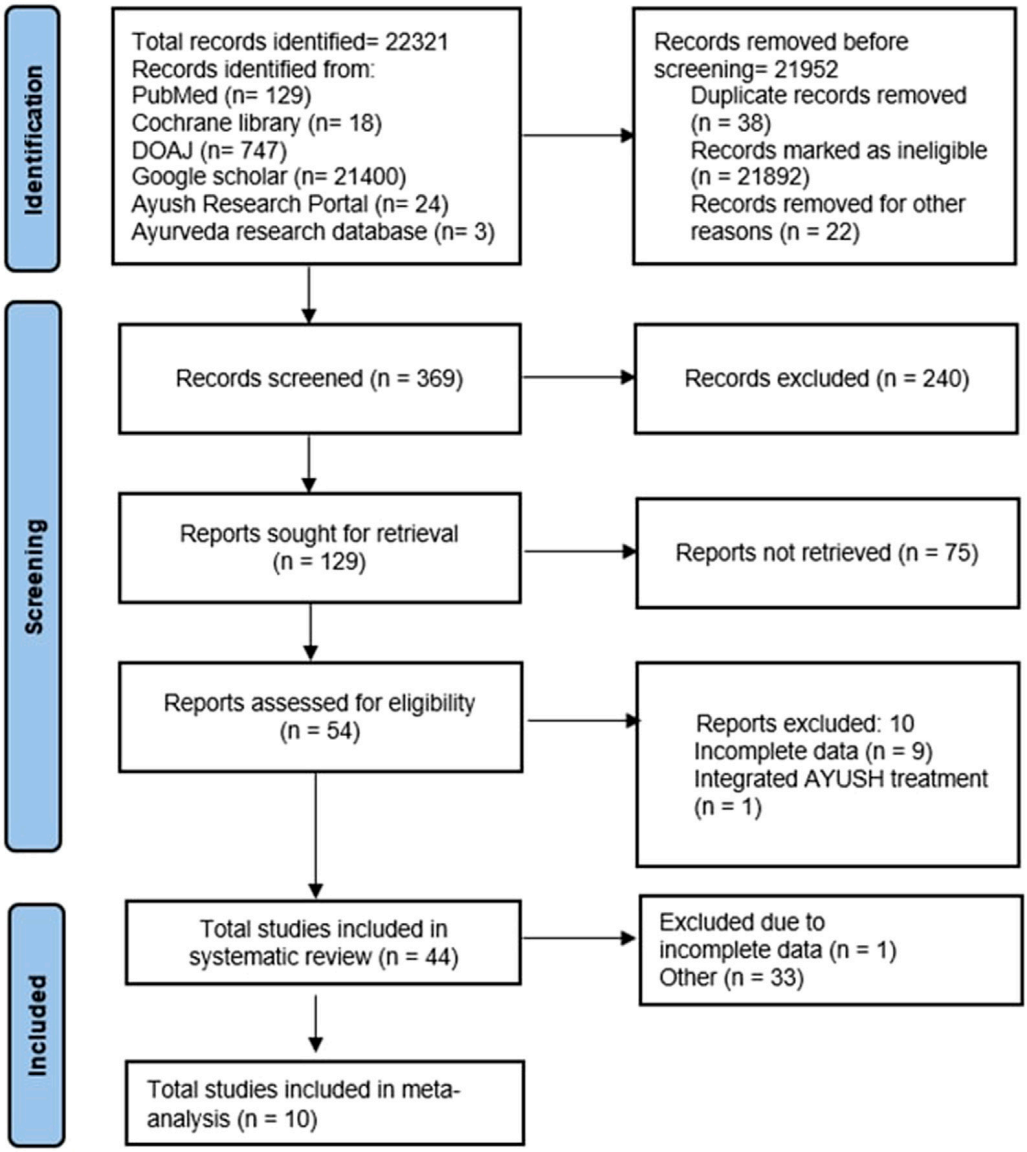
Flowchart showing the selection of studies for the review and meta-analysis.

A total of 22,321 studies were identified at the end of the preliminary search, of which we excluded 38 duplicate records, 21892 ineligible studies, and 22 other studies; the remaining 369 studies were screened for their titles and abstracts in the next step. Here, 129 studies with full texts were screened, and 44 studies were finally included in the systematic review based on the inclusion criteria after reading the full texts. Moreover, ten studies were included in the meta-analysis after excluding 34 studies owing to incomplete data. The literature screening process and results are illustrated in Figure 1.

### 3.2 Study characteristics and interventions

The 44 studies included in the systematic review are detailed in Tables 1–4. Of these, 15 were RCTs, 9 were single-arm studies, 17 were parallel-arm studies, and 3 were observational studies. All studies had consistent baselines and included both male and female subjects. The pre- and post-comparison data on the effects of Ayurvedic interventions on the SBP and DBP for both the intervention and control groups were used to determine inclusion. Ten of the fifteen RCTs were included in the meta-analysis and involved 118 participants in the Ayurveda vs. placebo category as well as 396 participants in the Ayurveda vs. active control category. The Ayurvedic interventions consisted of single drugs, polyherbal/herbomineral formulations, and/or *panchakarma.* The durations of intervention in the studies included in the systematic reviews varied from 10 days to 1 year. The details of each type of study are further explored below.

**TABLE 1:** Basic characteristics of the included single-arm studies.

Serial number	Authors (year)	Samples (total/completed)	Age (years)	Intervention	Dose/procedure time	Frequency	Duration	Outcome measures^a^	Adverse event	Outcome compared with baseline			
										↓SBP (MD)	*p*-value	↓DBP (MD)	*p*-value
1	Padhi et al. (2009)	127/113	35–70	*Brahmi yoga*	9 g/d	3 divided doses	90 days	1, 2, 3	Not mentioned	27.93	<0.001	15.21	<0.001
2	Gupta and Singh (1995)	35/35	>18	*Lekhana basti* (Shastri, 2017)	100 mL/d	8 days, once every 3 months	1 year	1, 3, 7, 8, 10	Not mentioned	11.14	<0.01	10.57	<0.001
3	Bharti and Bikshapati (2006)	25/22	35–70	*Arjuna vachadi yoga*	1 g/d	2 divided doses	3 months	1, 2	No adverse event	17.55	<0.001	14.64	<0.001
4	Sane et al. (2018)	30/30	30–70	Capsule artyl	1 g/d	2 divided doses	28 days	1	No adverse event	13.62	<0.001	0.68	>0.05
5	Kumawat et al. (2022)	150/100	18–50	Powder of *rudraksha* (*Elaeocarpus ganitrus* Roxb.; Elaeocarpaceae; Elaeocarpi ganitri semen)	2 g/d	2 divided doses	12 weeks	1, 2, 7	No adverse event	18.47	<0.001	8.26	<0.001
6	Hivale and Bhatted (2018)	15/15	20–60	*Virechana*	-	-	30 days	1, 2	No adverse event	28.67	<0.001	19.3	<0.001
7	Patel and Patel (2024)	30/28	20–50	*Virechana* + *Arjuna*, *Gokshura* (AG) powder	20 g/d	2 divided doses	15 days	1, 2	Not mentioned	19.29	<0.001	12.86	<0.001
8	Kar (2014)	12/12	>18	*Abhyanga*	30 min/d	Once a day	10 days	1, 2	No adverse event	17.33	<0.001	5.67	>0.05
9	Yadav et al. (2019)	25/25	30–60	Extract of *Eclipta alba* (L.) Hassk [Asteraceae; Ecliptae alba herba]	1 g/d	2 divided doses	30 days	1, 2, 3, 5, 6	No adverse event	16.1	<0.001	14.08	<0.001

^a^ 1-systolic and diastolic blood pressures (SBP, DBP), 2-clinical symptoms, 3-lipid profile, 4-liver function test, 5-kidney function test, 6-urine analysis, 7-pulse rate, 8-bodyweight, 9-quality of life, 10-neurohormone level, 11-serum electrolyte level, 12-angiotensin-converting enzyme (ACE) activity, 13-mean arterial pressure, 14-fibrinolytic activity, 15-Hamilton Anxiety Rating Scale (HARS) and/or Hamilton Rating Scale for Depression (HRSD). MD, mean difference.

**TABLE 2:** Basic characteristics of the included controlled trials.

Serial number	Authors (year)	Study type	Samples (total/completed)	Age (years)	Intervention	Dose	Frequency	Control	Duration	Outcome measures^a^	Adverse event	Outcome compared with baseline							
												Intervention				Control			
												↓SBP (MD)	*p*-value	↓DBP (MD)	*p*-value	↓SBP (MD)	*p*-value	↓DBP (MD)	*p*-value
1	Haji and Haji (1999)	RCT	80/54<	>18	Tea *of Hibiscus sabdariffa* Linn*.* [Malvaceae; Hibisci sabdariffae flos]	12 g/d	Once daily	No treatment	12 days	1	Not mentioned	17.61	<0.001	10.87	<0.001	6.26	<0.008	3.52	<0.02
2	Herrera-Arellano et al. (2004)	RCT	80/70<	30–80	Infusion of *Hibiscus sabdariffa* Linn*.* [Malvaceae; Hibisci sabdariffae flos]	20 g/d	2 divided doses	Captopril	4 weeks	1, 6	Not mentioned	14.15	<0.03	11.18	<0.06	16.43	<0.001	13.12	<0.001
3	Herrera-Arellano et al. (2007)	Double-blind RCT	193/171	25–61	Extract of *Hibiscus sabdariffa* Linn*.* [Malvaceae; Hibisci sabdariffae flos]	250 mg/d	Once daily	Lisinopril	4 weeks	1, 11, 12	HS: nervousness Control: dry cough, dry mouth	17.14	<0.001	11.97	<0.001	23.31	<0.001	15.39	<0.001
4	Jalalyazdi et al. (2019)	RCT	46/46	18–70	Tea *of Hibiscus sabdariffa* Linn*.* [Malvaceae; Hibisci sabdariffae flos] + non-medical treatment advice	2.5 g/d	2 divided doses	Non-medical treatment advice	30 days	1	Not mentioned	7.43	<0.001	6.7	<0.001	1.91	<0.004	3.96	<0.001
5	Ghaffari et al. (2020)	Triple-blind, placebo-controlled RT	92/81	18–80	Powder of *Emblica officinalis* Gaertn. [Phyllanthaceae; Emblicae officinalis fructus] + ongoing allopathic antihypertensive	1,500 mg/d	3 divided doses	Placebo + ongoing allopathic antihypertensive	8 weeks	1	No adverse event	26	<0.001	9.3	<0.001	11.8	<0.001	3.8	0.0014
6	Ashraf et al. (2013)	Single-blind, placebo-controlled study	210/192	20–70	Garlic extract	300 mg/d	Divided doses	Atenolol 50/100 mg	24 weeks	1	Two in placebo: abdominal discomfort, headache Three in garlic 1,500 mg/d: heartburn	2.3	<0.05	1.45	<0.05	9.2	<0.001	9.11	<0.001
					Garlic extract	600 mg/d	Divided doses	Placebo				4.3	<0.001	3.37	<0.001	0.2	<0.05	−1.04	<0.05
					Garlic extract	900 mg/d	Divided doses					6.1	<0.001	4.16	<0.001				
					Garlic extract	1,200 mg/d	Divided doses					6.7	<0.001	6.27	<0.001				
					Garlic extract	1,500 mg/d	Divided doses					7.6	<0.001	4.97	<0.001				
7	Sobenin et al. (2009)	Double-blind, placebo-controlled RT	90/84	35–70	Time-released garlic powder tablet (Allicor-1)	600 mg/d	2 divided doses	Placebo (identical)	8 weeks	1	Gastrointestinal complaints One in Allicor-2; one in the Kwai group	7	<0.001	3.8	<0.001	3.2	<0.001	1	<0.001
					Time-released garlic powder tablet (Allicor-2)	2,400 mg/d	4 Divided doses					9.3	<0.001	3.2	<0.001				
					Garlic powder (Kwai)	900 mg/d	3 Divided doses					5.4	<0.001	−1	0.08				
8	Dhawan and Jain (2004)	Double-blind, placebo-controlled RT	40/40	>18	Garlic pearls	Garlic oil 2.5% w/v	2 pearls/d	Placebo	8 weeks	1, 3	Gastrointestinal discomfort: 3 in garlic pearls and 2 in placebo	8	<0.05	9	<0.05	3	>0.05	2	>0.05
9	Panneerselvam et al. (2005)	Single-/double-blind RCT	57/57	35–70	*Ghana vati* with *Balsamodendron mukul* Hook*.* [Burseraceae; Commiphorae mukul guggulu]	*B. mukul* 1,500 mg/d	2 divided doses	Nifedipine 10 mg/day	6 weeks	1, 3	Not mentioned	20.23	<0.01	8.91	<0.05	20.88	<0.001	2.41	>0.05
						*B. mukul* 1,500 mg/d + nifedipine 10 mg/d		Control				26.83	<0.001	15.27	<0.01	1.95	>0.05	2.49	>0.05
10	Verma et al. (2012)	Single-blind, placebo-controlled RT	30/30	35–50	Powder of *Pueraria tuberosa* (*Willd*.) DC [Fabaceae (Leguminosae); Puerariae tuberosae tuber]	3 g	2 divided doses	Placebo	12 weeks	1, 14	Not mentioned	25	<0.001	11	<0.05	−3.9	>0.05	0.53	>0.05
11	Nwachukwu et al. (2015)	Double-blind RCT	78/75	31–70	Extract of *Hibiscus sabdariffa* Linn*.* [Malvaceae; Hibisci sabdariffae flos]	150 mg/kg/d	NA	Lisinopril 10 mg/d	4 weeks	1, 11, 12, 13	Lisinopril: cough in 3 cases Placebo: hypertension in 2 cases	17.08	<0.001	12.12	<0.001	12.6	<0.001	9.2	<0.001
								Placebo								1.1	>0.05	0.4	>0.05
12	Patil and Patel (2020)	RCT	60/60	30–70	*Karsha vati*	4 g/d	2 divided doses	Telmisartan 20 mg/d	6 weeks	1, 2	Not mentioned	6.13	<0.001	4.53	<0.001	12.2	<0.001	8.67	<0.001
13	Chaudhary and Rohila (2015)	RCT	45/40	60–90	Mixture of *Tagara* + *Gokshura* + *Triphala* powder	*Tagara* (2 g/d); *Gokshura* (12 g/d); *Triphala* (6 g/d)	*Tagara* and *Gokshura*: 2 divided doses *Triphala*: once daily	Amlodipine 5 mg/d	30 days	1, 3, 4, 5	No adverse event	16.4	<0.001	3	<0.001	24.5	<0.001	8.7	<0.001
14	Sharma et al. (2021)	Single-blind study	34/33	>18	*Shamak yoga*	4 g/d	2 divided doses	Atenolol 50 mg/d	4 weeks	1, 2	No adverse event	15.8	<0.001	10.7	<0.001	13.5	<0.001	12.3	<0.001
15	Nayak et al. (2015)	RCT	60/52	35–60	Extract of *punarnava*: *Boerhavia diffusa* Linn*.* [Nyctaginaceae; Boerhavia diffusa radix]	1 g/d	2 divided doses	Hydrochlorothiazide 12.5 mg	30 days	1, 11	Control: 23 cases Treatment: 3 cases	14.15	<0.001	8.3	<0.001	20.64	<0.001	9.68	<0.001

^a^ 1-systolic and diastolic blood pressures (SBP, DBP), 2-clinical symptoms, 3-lipid profile, 4-liver function test, 5-kidney function test, 6-urine analysis, 7-pulse rate, 8-bodyweight, 9-quality of life, 10-neurohormone level, 11-serum electrolyte level, 12-angiotensin-converting enzyme (ACE) activity, 13-mean arterial pressure, 14-fibrinolytic activity, 15-Hamilton Anxiety Rating Scale (HARS) and/or Hamilton Rating Scale for Depression (HRSD). RCT, randomized controlled trial; RT, randomized trial; MD, mean difference.

**TABLE 3:** Basic characteristics of the included comparative studies.

Serial number	Authors (year)	Samples (total/completed)	Age (years)	Intervention	Dose/procedure time	Frequency	Comparator	Dose	Frequency	Duration	Outcome measures^a^	Adverse event	Outcome compared with baseline							
													Intervention				Comparator			
													↓SBP (MD)	*p*-value	↓DBP (MD)	*p*-value	↓SBP (MD)	*p*-value	↓DBP (MD)	*p*-value
1	Bharathi and Swamy (2005)	75/66	35–70	*Arjuna vachadi yoga*	9 g/d	3 divided doses	CPS (*chandraprabha vati, punarnava mandora, sweta parpati*)	4.5 g/d	3 divided doses	6 weeks	1, 2	Not mentioned	22.91	<0.001	18.4	<0.001	19.38	<0.001	14.12	<0.001
2	Kundu et al. (2010)	47/40	>18	*Shirodhara* by *bala taila*	30 min	3 sessions for 7 days	Tablet of *sarpagandha* [*Rauwolfia serpentina* (L.) Benth.; Apocynaceae; Rauwolfia serpentina radix]	1 g/d	2 divided doses	30 days	1, 2	Not mentioned	8.3	<0.001	4.9	<0.001	5.3	<0.001	3	<0.001
3	Mishra and Tubaki (2019)	68/68	20–70	*Vati* capsule of *brahmi* [*Bacopa monnieri* (L.) Pennell; Plantaginaceae; Bacopa monnieri herba]	1 g/d	2 divided doses	*Ghana vati* of *sarpagandha* [*Rauwolfia serpentina* (L.) Benth.; Apocynaceae; Rauwolfia serpentina radix]	1 g/d	2 divided doses	30 days	1, 2, 3, 13	No adverse event	10.88	<0.001	2.85	0.01	12.83	<0.001	3.64	0.006
4	Shukla et al. (2013)	40/33	20–60	*Virechana + Arjunadi ghana vati* (AGV)	AGV 2 g/d	AGV 2 divided doses	*Basti +* AGV	16 *basti* + AGV 2 g/d	AGV 2 divided doses	30 days	1, 2	Not mentioned	21.75	<0.001	8.87	<0.02	20	<0.001	9.18	<0.001
5	Mishra et al. (2012)	20/20	>18	*Shankhapushpayadi ghana vati*	2 g/d	2 divided doses	*Ghana vati* of *sarpagandha* [*Rauwolfia serpentina* (L.) Benth.; Apocynaceae; Rauwolfia serpentina radix]	2 g/d	2 divided doses	8 weeks	1, 2	Not mentioned	14.4	<0.001	8.6	<0.001	19.6	<0.001	11.2	<0.01
6	Kylliang et al. (2019)	40/40	30–70	*Ashwagandhadi churna*	6 g/d	2 divided doses	Powder of *sarpagandha* [*Rauwolfia serpentina* (L.) Benth.; Apocynaceae; Rauwolfia serpentina radix]	2 g/d	2 divided doses	21 days	1, 2, 3	No adverse event	25.5	<0.001	8.8	0.132	30	<0.001	11.1	<0.001
7	Bhargavi and Chaithanya (2018)	60/60	>18	*Brahmi taila shirodhara*	NA	Once daily	*Jala shirodhara*	NA	Once daily	14 days	1, 13	Not mentioned	16.95	<0.001	12.7	<0.001	1.7	>0.05	1.9	>0.05
							*Tila taila shirodhara*										7.85	<0.001	4.35	<0.001
8	Gajraj et al. (2020)	40/40	>18	*Mansyadi yoga* (MY) + *Mansyadi kwath shirodhara* (MS)	MY: 2 g/d	MY: 2 divided doses MS: once daily	*Mansyadi yoga*	2 g/d	2 divided doses	14 days	1, 3, 4, 5	Not mentioned	23.6	<0.001	12.1	<0.001	20.2	<0.001	10.7	<0.001
9	Tanna et al. (2024)	103/103	20–65	Capsule BP norm	1 g/d	2 divided doses	Capsule BP norm + ongoing allopathic antihypertensive	500 mg/d	2 divided doses	4 weeks	1, 17	No adverse event	16.82	<0.001	13.71	<0.001	18.49	<0.001	13.95	<0.001
10	Prajapat et al. (2021)	40/40	18–70	*Tagaradi kwatha*	20 g/d	2 divided doses	*Tagaradi kwatha* (TK) + *shirodhara* (SD)	TK: 20 g/d	TK: 2 divided doses SD: once daily for 10 days	30 days	1, 2	No adverse event	19	<0.001	10	<0.001	23.9	<0.001	11.3	<0.001
11	Patil et al. (2021)	40/40	>18	*Mrudu samvahana* (forehead massage)	20 min/d	7 days	*Takradhara* (buttermilk dripping therapy)	40 min/d	7 days	15 days	1, 7	Not mentioned	22.2	<0.001	11.7	<0.001	29.4	<0.001	15.3	<0.001
12	Avhad et al. (2016)	102/91	25–65	*Raktadushtihar yoga*	4 g/d	2 divided doses	*Raktadushtihar yoga* + ongoing allopathic antihypertensive	4 g/d	2 divided doses	1 month	1, 2, 3, 4, 5	Not mentioned	19.57	<0.001	10.89	<0.001	22.07	<0.001	13.68	<0.001
13	Goyal and Rath (2020)	63/60	18–60	*Ghana vati* of polyherbal formulation	1 g/d	2 divided doses	Extract of polyherbal formulation	1 g/d	2 divided doses	90 days	1, 2, 3, 5	Not mentioned	14.33	<0.001	6.33	<0.001	17.03	<0.001	8.33	<0.001
14	Manju et al. (2020)	30/30	30–60	*Nitya virechana* with *trivrita churna*	10 g/d	Single dose	*Nitya virechana* with *aragvadha churna*	10 g/d	Single dose	30 days	1, 5, 11	No adverse event	9.2	<0.001	8.2	<0.001	10.47	<0.001	11.34	0.013
15	Murthy et al. (2000)	75/75	>18	*Gokshura* [*Tribulus terrestris*; Zygophyllaceae; Tribuli terrestris fructus] whole plant *ghanasatwa* (solid water extract)	3 g/d	3 divided doses	*Gokshura* [*Tribulus terrestris;* Zygophyllaceae; Tribuli terrestris fructus] fruit *ghanasatwa* (solid water extract)	3 g/d	3 divided doses	4 weeks	1, 2, 3, 7	No adverse event	18.66	<0.01	9.18	<0.01	17.41	<0.02	7.97	<0.02
							Control (lactose intraperitoneal administration)	-	-				-	-	-	-	2.68	>0.05	1.97	>0.05
16	Dhananjay (2003)	36/30	>18	*Medhya rasayana*	9 g/d	3 divided doses	*Kshiradhara* (milk dripping therapy)	45 min	-	2 months	1, 2, 3	Not mentioned	26.6	<0.001	11.6	<0.001	41.4	<0.001	14.4	<0.001
							*Medhya rasayana* + *kshiradhara*	-	-				-	-	-	-	32.4	<0.001	12.2	<0.001
17	Ramesh (2003)	44/32	>18	*Virechana* (V) + *shamana* (S)	V: As per *bala* and *kostha* S: 4.5 g/d	2 divided doses	*Shamana*	4.5 g/d	2 divided doses	1 month	1, 2, 3, 5	Not mentioned	36.47	<0.001	22.94	<0.001	26.4	<0.001	19.07	<0.001

^a^ 1-systolic and diastolic blood pressures (SBP, DBP), 2-clinical symptoms, 3-lipid profile, 4-liver function test, 5-kidney function test, 6-urine analysis, 7-pulse rate, 8-bodyweight, 9-quality of life, 10-neurohormone level, 11-serum electrolyte level, 12-angiotensin-converting enzyme (ACE) activity, 13-mean arterial pressure, 14-fibrinolytic activity, 15-Hamilton Anxiety Rating Scale (HARS) and/or Hamilton Rating Scale for Depression (HRSD). MD, mean difference.

**TABLE 4:** Basic characteristics of the included observational studies.

Serial number	Authors (year)	Samples (total/completed)	Age (years)	Intervention	Dose/procedure time	Frequency	Duration	Outcome measures^a^	Adverse event	Outcome compared with pre-intervention			
										Intervention			
										↓SBP (MD)	*p*-value	↓DBP (MD)	*p*-value
1	Nandha et al. (2011)	110/98	>18	Capsule *rakatchaphar*	1 g/d	2 divided doses	8 weeks	1	No adverse event	41.18	<0.001	20.9	<0.001
2	Ali et al. (2015)	40/40	30–70	*Brahmyadi churna* + tablet *shilajatu* (purified asphaltum)	18 g/d	3 divided doses	30 days	1	No adverse event	22.7	<0.001	14.6	<0.001
3	Pradeep et al. (2014)	30/30	30–70	*Shirodhara* + tablet arjin	40 min + 1 tablet	Thrice daily	30 days	1	Not mentioned	23.5	<0.001	17.6	<0.001

^a^ 1-systolic and diastolic blood pressures (SBP, DBP), 2-clinical symptoms, 3-lipid profile, 4-liver function test, 5-kidney function test, 6-urine analysis, 7-pulse rate, 8-bodyweight, 9-quality of life, 10-neurohormone level, 11-serum electrolyte level, 12-angiotensin-converting enzyme (ACE) activity, 13-mean arterial pressure, 14-fibrinolytic activity, 15-Hamilton Anxiety Rating Scale (HARS) and/or Hamilton Rating Scale for Depression (HRSD). MD, mean difference.

#### 3.2.1 Single-arm studies

Table 1 shows the details of nine single-arm studies that evaluated the impacts of various Ayurvedic interventions on BP. These interventions included classical therapies like *Virechana* (purgation therapy), *Abhyanga* (oil massage), and herbal formulations like *Eudraksha churna*, *Arjuna vachadi yoga*, and *Eclipta alba* (*Bhringaraj*) extract. Most of these studies demonstrated significant reductions in both SBP and DBP, with *p*-values < 0.001 in nearly all cases. Although these studies indicate strong therapeutic potential, their lack of control groups limits the inference on causality.

#### 3.2.2 Controlled trials

Table 2 shows the details of the 15 RCTs included in the systematic review that compared Ayurvedic interventions to standard care, placebo, or no treatment. Many of these trials utilized *Hibiscus sabdariffa* (*ambasthaki*) tea or extracts and found substantial BP-lowering effects. Similarly, Ghaffari et al. (2020) demonstrated that *Emblica officinalis* (*Amalaki*) combined with conventional drugs significantly outperformed placebo. Garlic-based preparations were evaluated extensively across multiple arms in trials reported by Ashraf et al. (2013) and Sobenin et al. (2009) that confirmed dose-dependent antihypertensive effects. The other notable herbs included *Boerhavia diffusa* (*Punarnava*) extract, *Balsamodendron mukul* (*Guggulu*), and the herbal formulation *Karsha vati*, all of which showed statistically significant improvements in BP outcomes. These trials provide valuable comparative insights, although blinding and allocation concealment were inconsistently reported.

#### 3.2.3 Comparative studies

Table 3 shows the details of 17 comparative studies that analyzed Aurvedic formulations against other Ayurvedic treatments. For example, Bharathi and Swamy (2005) found that *Arjuna vachadi yoga* (a polyherbal formulation) and another polyherbal formulation with *Chandra prabhavati, Punarnava mandora,* and *Sweta parpati* (CPS) significantly reduced BP, with slightly better outcomes in the *Arjuna vachadi yoga* group. *Panchakarma* therapies like *Virechana*, *Shirodhara* (oil dripping on forehead), and *Takradhara* (buttermilk dripping on forehead) were also found to feature prominently in combination with herbal therapies. Combination interventions like *virechana* and *Arjunadi ghana vati* demonstrated significant reductions in SBP and DBP compared to control modalities like *Basti* or *Sarpagandha vati*. Avhad et al. (2016) showed that combining *Baktadushtihar yoga* with conventional antihypertensives improved outcomes over standalone therapy. These comparative studies emphasize the synergy between procedural and pharmacological Ayurvedic approaches but are constrained by the heterogeneity in comparators and short study durations.

#### 3.2.4 Observational studies

Table 4 shows the details of three observational studies that documented BP outcomes following Ayurvedic treatments without control groups. Nandha et al. (2011) reported remarkable reductions in the SBP and DBP after 8 weeks of *Rakatchaphar* capsule use. Similarly, Ali et al. (2015) and Pradeep et al. (2014) demonstrated strong BP-lowering effects with *Brahmyadi churna* and *Shilajatu* as well as *shirodhara* and arjin tablet, respectively.

### 3.3 Risk of bias

**FIGURE 2:**
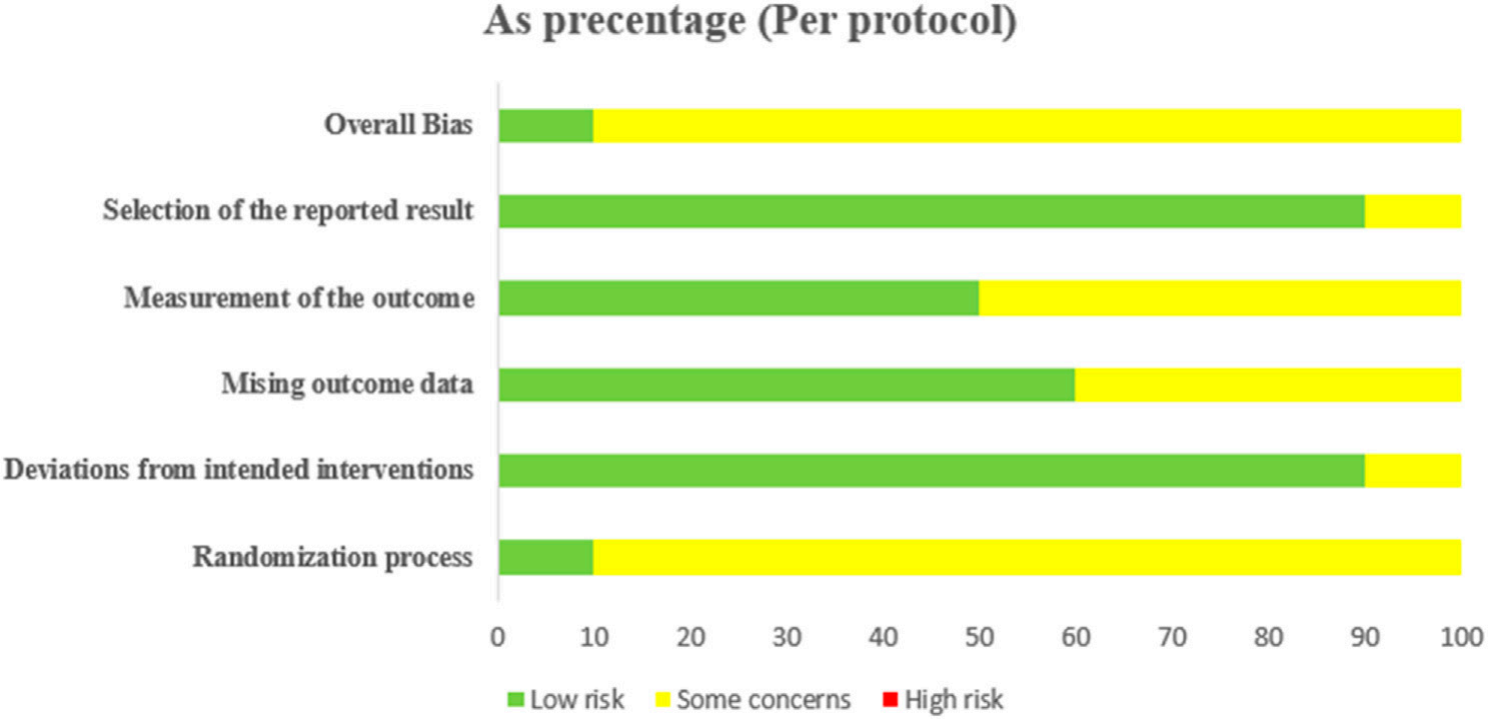
Graph depicting the risk of bias items.

**FIGURE 3:**
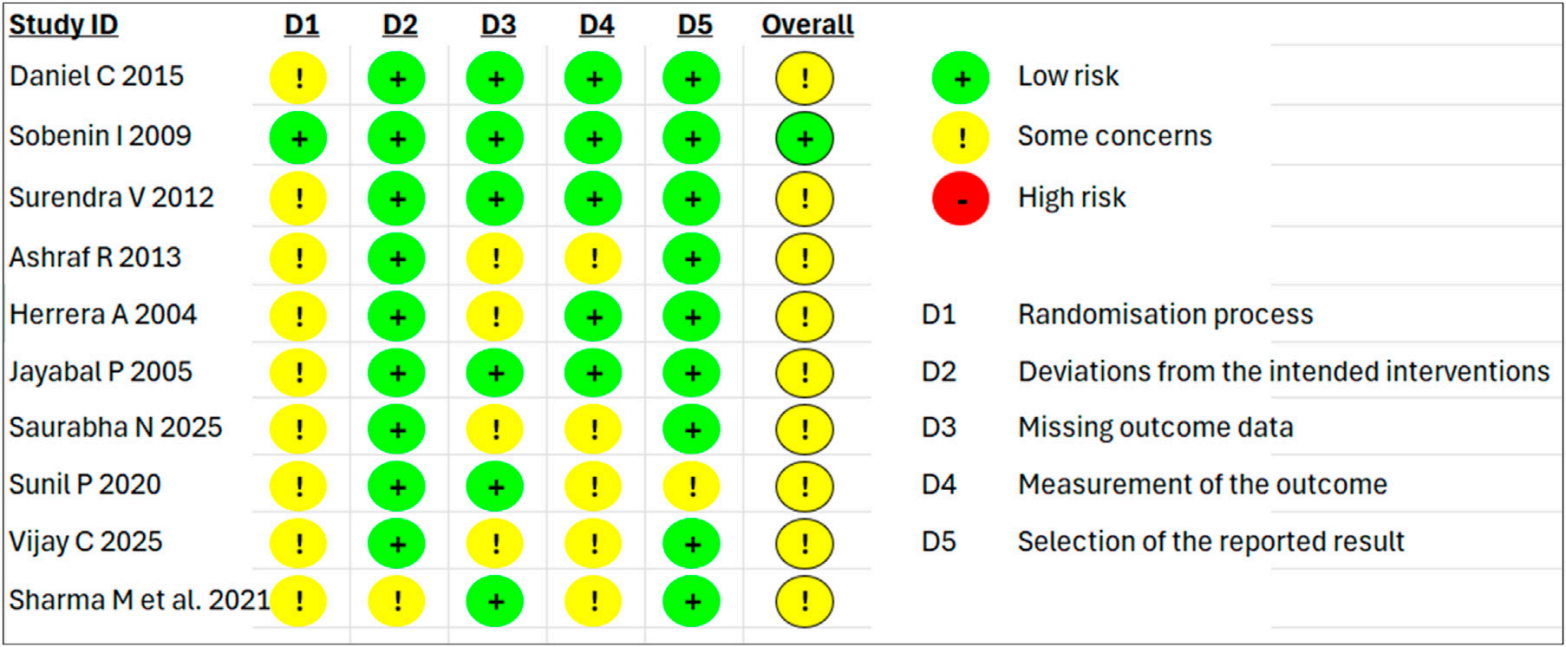
Summary of the risk of bias items and their levels of concern in different studies.

The risk of bias evaluation was performed on the ten RCTs included in the meta-analysis. This assessment is summarized in Figure 2 that shows the risk of bias graph (the reviewers’ judgments about each of the items are presented as percentages across all included studies) and Figure 3 that shows the risk of bias summary based on the reviewers’ judgment about each item of each study.

#### 3.3.1 Randomization process

The ten studies included in these assessments had no baseline differences between the intervention groups, which is a sign of good randomization; however, all studies lacked allocation concealment. Among these works, the trial reported by Sobenin et al. (2009) was the only one with a low risk of bias.

#### 3.3.2 Deviations from the intended interventions

Seven of the ten studies reported blinding (Herrera-Arellano et al., 2004; Ashraf et al., 2013; Sobenin et al., 2009; Panneerselvam et al., 2005; Verma et al., 2012; Nwachukwu et al., 2015; Sharma et al., 2021), whereas the remaining three studies did not report blinding (Patil and Patel, 2020; Chaudhary et al., 2015; Nayak et al., 2015); Most studies reported a number of participants analysed with documented reasons for dropped outs except that of Sharma et al. (2021) were judged to have a low risk of bias.

#### 3.3.3 Missing outcome data

The SBP and DBP data were available for all or nearly all participants randomized in six of the ten studies (Nwachukwu et al., 2015; Sobenin et al., 2009; Verma et al., 2012; Panneerselvam et al., 2005; Patil and Patel, 2020; Sharma et al., 2021), representing low risk of bias, whereas the remaining four studies reported some concerns due to attrition (Herrera-Arellano et al., 2004; Ashraf et al., 2013; Chaudhary et al., 2015; Nayak et al., 2015).

#### 3.3.4 Measurement of outcome

Five of the ten studies reported appropriate methods of outcome measurement (Nwachukwu et al., 2015; Sobenin et al., 2009; Verma et al., 2012; Herrera-Arellano et al., 2004; Panneerselvam et al., 2005) and were thus judged to have a low risk of bias, whereas the remaining five studies reported some concerns owing to lack of blinding of the outcome assessors (Sharma et al., 2021; Nayak et al., 2015; Ashraf et al., 2013; Patil and Patel, 2020; Chaudhary et al., 2015).

#### 3.3.5 Selection of the reported result

Nine of the ten studies presented results that were obtained using prespecified analysis plans (Nwachukwu et al., 2015; Verma et al., 2012; Sobenin et al., 2009; Ashraf et al., 2013; Herrera-Arellano et al., 2004; Panneerselvam et al., 2005; Chaudhary et al., 2015; Sharma et al., 2021; Nayak et al., 2015).

#### 3.3.6 Overall bias

Based on the five evaluation items discussed above, nine of the ten studies (Nwachukwu et al., 2015; Verma et al., 2012; Ashraf et al., 2013; Herrera-Arellano et al., 2004; Panneerselvam et al., 2005; Chaudhary et al., 2015; Sharma et al., 2021; Nayak et al., 2015; Patil and Patel, 2020) showed concerns due to attrition rate, lack of randomization, blinding, etc.

### 3.4 Effectiveness assessment

We performed a meta-analysis using a total of ten RCTs that were further divided into two groups as Ayurveda vs. placebo and Ayurveda vs. active control.

#### 3.4.1 Ayurveda vs. placebo

**FIGURE 4:**
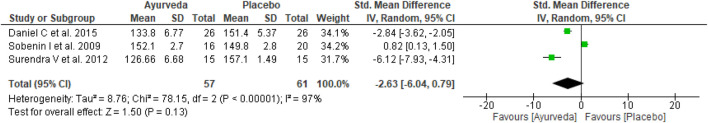
Standardized mean differences (MDs) for systolic blood pressure (Ayurveda vs. placebo).

**FIGURE 5:**
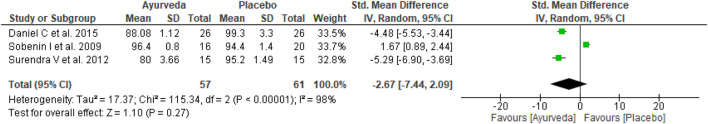
Standardized MDs for diastolic blood pressure (Ayurveda vs. placebo).

Three of the studies included in the meta-analysis (Nwachukwu et al., 2015; Sobenin et al., 2009; Verma et al., 2012) compared the effects of Ayurvedic interventions with those of placebo on EH. The forest plot of these studies illustrates the range of SBP and DBP values observed in the analysis. The pooled estimates show non-significant effects of the Ayurvedic interventions on the SBP (n = 118; MD = −2.63 mmHg, 95% CI = −6.04 to 0.79, *p* = 0.13; heterogeneity: I^2^ = 97%, χ^2^ = 78.15, *p* < 0.00001) (Figure 4) and DBP (n = 118; MD = −2.67 mmHg, 95% CI = −7.44 to 2.09, *p* = 0.27; heterogeneity: I^2^ = 98%, χ^2^ = 115.34, *p* < 0.00001) (Figure 5) compared to placebo. Although the effects were non-significant owing to high heterogeneity, two of the three trials favored Ayurveda over placebo.

#### 3.4.2 Ayurveda vs. active control

**FIGURE 6:**
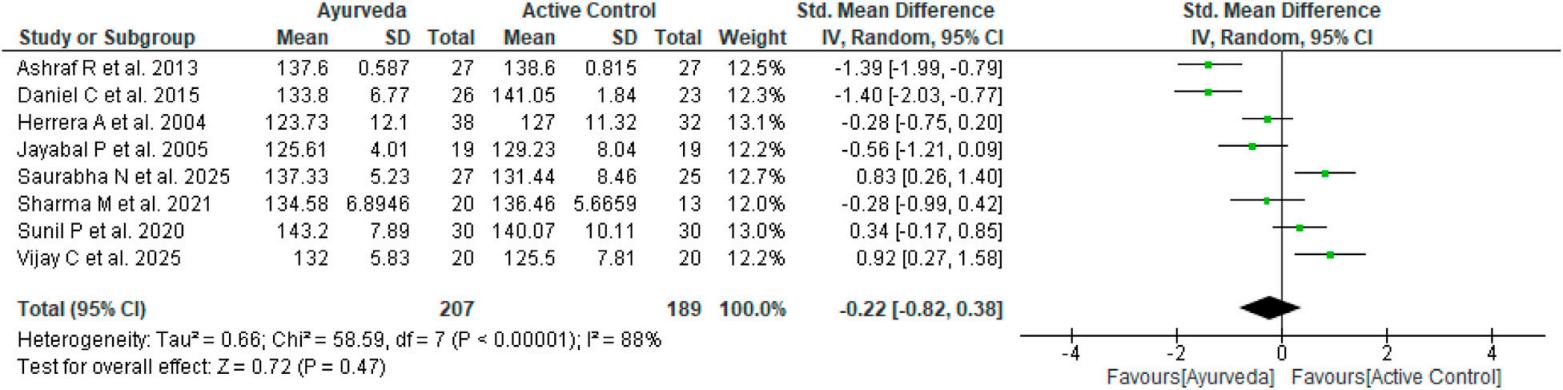
Standardized MDs for systolic blood pressure (Ayurveda vs. active control).

**FIGURE 7:**
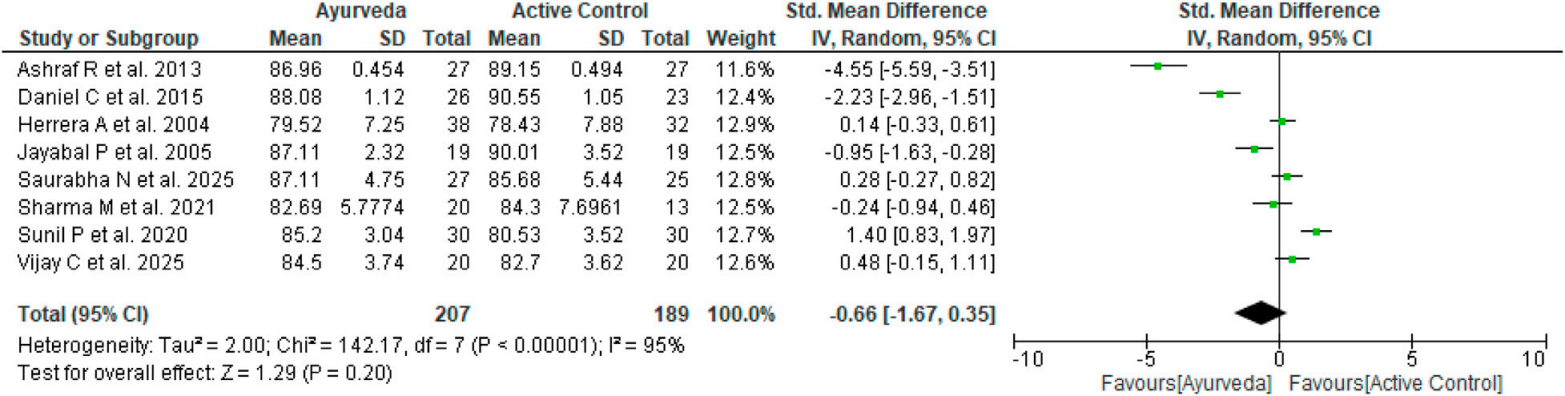
Standardized MDs for diastolic blood pressure (Ayurveda vs. active control).

Eight of the ten studies included in the meta-analysis (Ashraf et al., 2013; Nwachukwu et al., 2015; Herrera-Arellano et al., 2004; Panneerselvam et al., 2005; Nayak et al., 2015; Sharma et al., 2021; Patil and Patel, 2020; Chaudhary et al., 2015) compared the effects of Ayurvedic interventions with those of antihypertensive drugs on EH. The forest plot of these studies illustrates the range of SBP and DBP values observed in the analysis. The meta-analysis revealed non-significant effects of the Ayurvedic interventions on the SBP (n = 396; MD = −0.22 mmHg, 95% CI = −0.82 to 0.38, *p* = 0.47; heterogeneity: I^2^ = 88%, χ^2^ = 58.59, *p* < 0.00001) (Figure 6) and DBP (n = 396; MD = −0.66 mmHg, 95% CI = −1.67 to 0.35, *p* = 0.20; heterogeneity: I^2^ = 95%, χ^2^ = 142.17, *p* < 0.00001) (Figure 7) compared to active control. Although the effects were non-significant owing to high heterogeneity, five of these eight trials favored Ayurveda over standard antihypertensive treatment.

##### 3.4.3 Adverse events

The adverse events associated with the Ayurvedic interventions were also evaluated for the included studies. Of the 44 works analyzed in this review, 17 studies reported “no adverse events” narratively (Bharti et al., 1999; Sane et al., 2018; Kumawat et al., 2022; Hivale and Bhatted, 2018; Kar, 2014; Yadav et al., 2019; Ghaffari et al., 2020; Chaudhary et al., 2015; Sharma et al., 2021; Mishra and Tubaki, 2019; Kylliang et al., 2019; Tanna et al., 2024; Prajapat et al., 2021; Manju et al., 2020; Murthy et al., 2000; Nandha et al., 2011; Ali et al., 2015), while 21 studies did not mention anything about adverse effects (Padhi et al., 2009; Gupta and Singh et al., 1995; Patel and Patel et al., 2024; Haji and Haji et al., 1999; Herrera-Arellano et al., 2004; Jalalyazdi et al., 2019; Panneerselvam et al., 2005; Verma et al., 2012; Patil and Patel, 2020; Bharathi and Swamy, 2005; Kundu et al., 2010; Shukla et al., 2013; Mishra et al., 2012; Bhargavi and Chaithanya, 2018; Gajraj et al., 2020; Patil et al., 2021; Avhad et al., 2016; Goyal and Rath, 2020; Dhananjay, 2003; Ramesh, 2003; Pradeep et al., 2014). A total of 33 adverse events were reported for the control group, of which four were attributed to Lisinopril (dry cough, dry mouth) (Herrera-Arellano et al., 2007; Nwachukwu et al., 2015), 23 were attributed to Hydrochlorothiazide (muscle pain, anorexia, constipation, and excess thirst) (Nayak et al., 2015), and six were attributed to the placebo (gastrointestinal discomfort, headache) (Ashraf et al., 2013; Dhawan and Jain, 2004; Sobenin et al., 2009). A total of 14 adverse events were reported in the Ayurvedic intervention group, of which two instances were attributable to the *H. sabdariffa* extract (nervousness) (Herrera-Arellano et al., 2007), three were attributable to the highest dose (1,500 mg/d) of garlic extract (heartburn) (Ashraf et al., 2013), two were attributable to the highest dose (2,400 mg/d) of Allicor and one in Kwai group (Sobenin et al., 2009), three were attributable to garlic pearls (gastrointestinal discomfort) (Dhawan and Jain, 2004), and three were attributable to *B. diffusa* extract (Nayak et al., 2015). No adverse events were noted in the groups receiving interventions in the classical forms, such as powder and decoction.

## 4 Discussion

This systematic review with meta-analysis presents a thorough assessment of the impacts of various Ayurvedic interventions on the SBP and DBP metrics in individuals with EH. A total of 44 studies were included in the systematic review, of which ten RCTs were found to be suitable for the meta-analysis. These studies encompass a wide array of Ayurvedic practices, including administration of single herbs, polyherbal formulations, *Panchakarma* therapies (e.g., *Virechana* and *Basti*), and combinations with other modalities like *shirodhara*.

The aggregated results from the RCTs comparing Ayurvedic to placebo interventions revealed no statistically significant reductions in the SBP or DBP, although the trends favored Ayurveda. However, the high heterogeneity (I^2^ > 95%) among the studies undermines the reliability of these findings; this heterogeneity may be attributed to variations in the herbal formulations, dosages, intervention durations, and participant characteristics. Additionally, the lack of placebo standardization and small sample sizes limited the statistical power and internal validity of the studies.

Similarly, when the Ayurvedic interventions were evaluated against standard antihypertensive treatments, the meta-analysis showed no significant differences in the SBP or DBP. Nonetheless, several individual studies documented clinically significant reductions in BP with Ayurvedic therapies that were comparable to the effects achieved with modern pharmacotherapy. These findings suggest the non-inferiority potential of Ayurveda that may have implications for patients seeking holistic or alternative care, particularly in cases where modern medications are contraindicated or poorly tolerated.

Numerous studies have also identified the advantages of Ayurveda on the lipid profile, liver and kidney functions, neurohormonal balance, and quality of life indicators, which are not considered in the current meta-analysis but warrant further investigations (Patel and Patel, 2024; Ghaffari et al., 2020; Sobenin et al., 2009; Bharathi and Swamy, 2005). These pleiotropic effects are consistent with the holistic pathophysiological framework of Ayurveda, which prioritizes multitarget strategies and systemic equilibrium over isolated symptomatic relief. Notably, no significant adverse effects were reported in any of the studies, underscoring the potential safety of Ayurvedic interventions when applied appropriately.

The risk of bias assessment indicated that although randomization was generally adequate, several of the studies lacked allocation concealment and blinding, particularly with regard to the outcome assessors. This drawback introduces the potential for performance and detection biases. Additionally, selective reporting and insufficient methodological transparency undermine confidence in these results.

Adverse events were found more often with the standard antihypertensive drugs used in the active control group than the Ayurvedic interventions. All of the adverse events reported for the Ayurvedic interventions were associated with extracts of single drugs and mostly at their high doses. Additionally, there were no notable adverse events for interventions administered in their classical forms, such as powder, decoction, and tablet. Hence, further studies are needed to evaluate the safety of classical Ayurvedic formulations compared to extracts of single drugs.

Given the global prevalence of hypertension and the associated challenges with adherence, side effects, and accessibility of conventional antihypertensive treatments, we propose that Ayurveda may offer a viable integrative or complementary approach to the management of hypertension. This is particularly relevant in low-resource settings or among populations that favor traditional medical systems. Nevertheless, the current body of evidence is limited by methodological constraints, including small sample sizes, lack of blinding, lack of allocation concealment, short intervention durations, and selective reporting.

This review demonstrates several strengths, including strict adherence to PRISMA guidelines, a comprehensive literature search encompassing both mainstream and Ayurvedic databases, and well-defined inclusion and exclusion criteria. However, the review is limited by the predominance of single-center studies, small sample sizes, non-uniform outcome measures, and short intervention durations.

### 4.1 Recommendations for future research

Given our findings in this survey, we offer some recommendations for future research. To substantiate the efficacy and safety of specific Ayurvedic interventions, it is imperative to conduct larger multicenter double-blind RCTs by employing rigorous methodologies. Long-term follow-up studies are also crucial to assess the sustainability of BP control and to evaluate the cardiovascular outcomes. Furthermore, the development of standardized protocols for Ayurvedic treatments and reporting guidelines would enhance the quality of evidence and facilitate cross-study comparisons. To fortify the evidence base, there is a pressing need for rigorously designed, adequately powered, multicenter RCTs with standardized intervention protocols, validated outcome measures, and extended follow-up periods. Such studies should also investigate the mechanistic insights, pharmacodynamic interactions, and comparative efficacies of Ayurvedic interventions against or as add-ons to contemporary antihypertensive therapies.

## 5 Conclusion

This systematic review and meta-analysis involved evaluation of the effectiveness and safety of Ayurvedic interventions in EH. Although the pooled results from the RCTs did not show statistically significant reductions in the SBP or DBP metrics compared to placebo or standard antihypertensive therapies, several individual studies have demonstrated clinically meaningful effects favoring Ayurvedic drugs. These interventions were generally well tolerated, with fewer and milder adverse events observed at higher doses of single-drug extracts than conventional medications. However, the overall certainty of evidence is limited by the high heterogeneity, small sample sizes, and methodological shortcomings in the included studies. Hence, well-designed, large-scale, and multicenter randomized trials with standardized protocols are required to establish the efficacies, safeties, and long-term benefits of Ayurvedic interventions in the management of hypertension.

## Data Availability

The original contributions presented in the study are included in the article/Supplementary Material, further inquiries can be directed to the corresponding author.
