# Supplementary material for: Effectiveness and safety of Ayurvedic intervention in essential hypertension: a systematic review with meta-analysis
Source: Front Pharmacol. 2025 Dec 19;16:1695614. doi: 10.3389/fphar.2025.1695614 (PMC12757693; doi:10.3389/fphar.2025.1695614)
Supplement: Supplementary file 1 [file Supplementaryfile1.docx]

**Appendix 1:**

**Search strategy**

| **Intervention** | **Disorder** |
| --- | --- |
| Ayurved* | Primary hypertension |
| Ayurvedic Medicine | Essential hypertension |
| Hindu medicine | Idiopathic hypertension |
| Ayurveda intervention |  |
| Ayurveda therapy |  |
| Panchkarma |  |
| Herbal medicine |  |
| Ayurveda plants |  |
| Polyherbal formulation |  |

**Appendix 2:**

**Contents of polyherbal formulations**

| **SN** | **Formulation Name /**  **Therapy** | **Author, Year** | **Content (Ayurveda Name)** | **Content (Latin Name – Standard Format)** |
| --- | --- | --- | --- | --- |
| *1.* | *Brahmi Yoga* | Padhi M et al. (2009) | *Brahmi*  *Vacha*  *Jatamansi*  *Arjuna* | *Bacopa monnieri* (L.) Pennell  [Plantaginaceae; Bacopae monnieri herba]  *Acorus calamus* Linn.  [Acoraceae; Acori calami rhizoma]  *Nardostachys jatamansi* DC.  [Caprifoliaceae; Nardostachyos rhizoma et radix]  *Terminalia arjuna* Roxb.  [Combretaceae; Terminaliae arjunae cortex] |
| *2.* | *Arjuna Vacadi Yoga* | Bharti et al. (1999);  Bharathi K et al. (2005) | *Arjuna*  *Vacha*  *Brahmi*  *Jatamansi* | *Terminalia arjuna* Roxb.  [Combretaceae; Terminaliae arjunae cortex]  *Acorus calamus* Linn.  [Acoraceae; Acori calami rhizoma]  *Bacopa monnieri* (L.)  [Plantaginaceae; Bacopae monnieri herba]  *Nardostachys jatamansi* DC.  [Caprifoliaceae; Nardostachyos rhizoma et radix] |
| 3. | Capsule Artyl | Sane R et al. (2018) | *Brahmi*  *Shunthi* | *Bacopa monnieri* (L.) Pennell  [Plantaginaceae; Bacopae monnieri herba]  *Zingiber officinale* Roscoe  [Zingiberaceae; Zingiberis rhizoma] |
| *4.* | *Arjuna– Gokshura Churna* | Patel R et al. (2024) | *Arjuna*  *Gokshura* | *Terminalia arjuna* Roxb.  [Combretaceae; Terminaliae arjunae cortex]  *Tribulus terrestris* L.  [Zygophyllaceae; Tribuli terrestris fructus] |
| *5.* | *Karsha Vati* | Sunil P *et* *al*. (2020) | *Ashwagandha*  *Bibhitaki* | *Withania somnifera* (L.) Dunal  [Solanaceae; Withaniae somniferae radix]  *Terminalia bellirica* (Gaertn.) Roxb. [Combretaceae; Terminaliae belliricae fructus] |
| *6.* | *Tagar– Gokshura–Triphala Churna* | Vijay C et al. (2025) | *Tagar*  *Gokshura,*  *Haritaki*  *Bibhitaki,*  *Amalaki* | *Valeriana wallichii* DC.  [Caprifoliaceae; Valerianae wallichii radix]  *Tribulus terrestris* L.  [Zygophyllaceae; Tribuli terrestris fructus]  *Terminalia chebula* Retz.  [Combretaceae; *Terminaliae chebulae fructus*]  *Terminalia bellirica* (Gaertn.) Roxb. [Combretaceae; *Terminaliae belliricae fructus*]  *Emblica officinalis* Gaertn.  [Phyllanthaceae; *Emblicae officinalis fructus*] |
| *7.* | *Shamak Yoga* (SY) | Sharma M *et* *al*. (2021) | *Vacha*  *Shati*  *Tagar*  *Pushkarmool*  *Ela*  *Ashwagandha*  *Brahmi*  *Shankhapushpi*  *Jatamansi*  *Musta* | *Acorus calamus* Linn.  [Acoraceae; Acori calami rhizoma]  *Hedychium spicatum* Sm.  [Zingiberaceae; Hedychii spicati rhizoma]  *Valeriana wallichii* DC.  [Caprifoliaceae; Valerianae wallichii radix]  *Inula racemosa* Hook.f.  [Asteraceae; Inulae racemosae radix]  *Elettaria cardamomum* (L.) Maton  [Zingiberaceae; Elettariae cardamomi fructus]  *Withania somnifera* (L.) Dunal  [Solanaceae; Withaniae somniferae radix]  *Bacopa monnieri* (L.) Pennell  [Plantaginaceae; Bacopae monnieri herba]  *Convolvulus pluricaulis* Choisy  [Convolvulaceae; Convolvuli pluricaulis herba*]*  *Nardostachys jatamansi* DC.  [Caprifoliaceae; Nardostachyos rhizoma et radix]  *Cyperus rotundus* L.  [Cyperaceae; Cyperi rotundi rhizoma] |
| *8.* | *Shankhapushpyadi Ghana Vati* | Jyoti M et al. (2012) | *Shankhapushpi*  *Brahmi*  *Guduchi*  *Aragvadha*  *Nimba*  *Kushta*  *Vacha*  *Gokshura* | *Convolvulus pluricaulis* Choisy  [Convolvulaceae; Convolvuli pluricaulis herba]  *Bacopa monnieri* (L.) Pennell  [Plantaginaceae; Bacopae monnieri herba]  *Tinospora cordifolia* (Willd.) Miers [Menispermaceae; Tinosporae cordifoliae caulis]  *Cassia fistula* Linn.  [Fabaceae; Cassiae fistulae fructus]  *Azadirachta indica* A. Juss.  [Meliaceae; Azadirachtae indicae folium]  *Saussurea lappa* C.B. Clarke  [Asteraceae; Saussureae lappae radix]  *Acorus calamus* Linn.  [Acoraceae; Acori calami rhizoma]  *Tribulus terrestris* Linn.  [Zygophyllaceae; Tribuli terrestris fructus] |
| *9.* | *Sarpagandhadi Ghana Vati* | Jyoti M et al. (2012) | *Sarpagandha*  *Jatamansi*  *Parseek Yavani*  *Pippalimoola* | *Rauvolfia serpentina* (L.) Benth. ex Kurz  [Apocynaceae; Rauvolfiae serpentinae radix]  *Nardostachys jatamansi* DC.  [Caprifoliaceae; Nardostachyos radix et rhizoma]  *Hyoscyamus niger* Linn.  [Solanaceae; Hyoscyami herba]  *Piper longum* Linn.  [Piperaceae; Piperis longi radix] |
| *10.* | *Ashwagandhadi Churna* | Aitilin K et al. (2019) | *Ashwagandha*  *Shunthi*  *Pippali*  *Maricha*  *Ela*  *Vidanga*  *Nagakeshara* | *Withania somnifera* (L.) Dunal  [Solanaceae; Withaniae somniferae radix]  *Zingiber officinale* Roscoe  [Zingiberaceae; Zingiberis rhizoma]  *Piper longum* Linn.  [Piperaceae; Piperis longi fructus]  *Piper nigrum* Linn.  [Piperaceae; Piperis nigri fructus]  *Elettaria cardamomum* (L.) Maton  [Zingiberaceae; Elettariae cardamomi semen]  *Embelia ribes* Burm. f.  [Primulaceae; Embeliae ribis fructus]  *Mesua ferrea* Linn.  [Calophyllaceae; Mesuae ferreae flos] |
| 11. | *Mansyadi Yoga* | Gajraj V et al. (2020) | *Jatamansi*  *Shankhapushpi*  *Sarpagandha*  *Arjuna*  *Tagar*  *Pippalimoola*  *Haritaki*  *Bibhitaki*  *Amalaki*  *Shunthi*  *Maricha*  *Pippali* | *Nardostachys jatamansi* DC.  [Caprifoliaceae; Nardostachyos rhizoma et radix]  *Convolvulus pluricaulis* Choisy  [Convolvulaceae; Convolvuli pluricaulis herba]  *Rauvolfia serpentina* (L.) Benth. ex Kurz  [Apocynaceae; Rauvolfiae serpentinae radix]  *Terminalia arjuna* Roxb. ex DC.  [Combretaceae; Terminaliae arjunae cortex]  *Valeriana wallichii* DC.  [Caprifoliaceae; Valerianae wallichii radix]  *Piper longum* Linn.  [Piperaceae; Piperis longi radix]  *Terminalia chebula* Retz.  [Combretaceae; Terminaliae chebulae fructus]  *Terminalia bellirica* (Gaertn.) Roxb.  [Combretaceae; Terminaliae belliricae fructus]  *Phyllanthus emblica* L.  [Phyllanthaceae; Emblicae officinalis fructus]  *Zingiber officinale* Roscoe  [Zingiberaceae; Zingiberis rhizoma]  *Piper nigrum* L.  [Piperaceae; Piperis nigri fructus]  *Piper longum* L.  [Piperaceae; Piperis longi fructus] |
| 12. | *Mansyadi* *Kwath* | Gajraj V et al. (2020) | *Jatamansi*  *Ashwagandha*  *Parasika Yavani* | *Nardostachys jatamansi* DC.  [Caprifoliaceae; Nardostachyos rhizoma et radix]  *Withania somnifera* (L.) Dunal  [Solanaceae; Withaniae somniferae radix]  *Hyoscyamus niger* Linn.  [Solanaceae; Hyoscyami nigri folium] |
| 13. | Capsule BP Norm | Ila T et al. (2024) | *Bhringaraja*  *Haritaki*  *Ashwagandha*  *Shankhapushpi*  *Sarpagandha* | *Eclipta alba* (L.) Hassk.  [Asteraceae; Ecliptae albae herba]  *Terminalia chebula* Retz.  [Combretaceae; Terminaliae chebulae fructus]  *Withania somnifera* (L.) Dunal  [Solanaceae; Withaniae somniferae radix];  *Convolvulus pluricaulis* Choisy  [Convolvulaceae; Convolvuli pluricaulis herba]  *Rauvolfia serpentina* (L.) Benth. ex Kurz  [Apocynaceae; Rauvolfiae serpentinae radix] |
| 14. | *Tagaradi Kwatha* | Preeti P et al. (2021) | *Tagara*  *Ashwagandha*  *Parpataka*  *Shankhapushpi*  *Devadaru*  *Kutaki*  *Brahmi*  *Jatamansi*  *Mustaka*  *Aragvadha*  *Haritaki*  *Draksha* | *Valeriana wallichii* DC.  [Caprifoliaceae; Valerianae wallichii radix]  *Withania somnifera* (L.) Dunal  [Solanaceae; Withaniae somniferae radix]  *Fumaria vaillantii* Loisel.  [Papaveraceae; Fumariae herba]  *Convolvulus pluricaulis* Choisy  [Convolvulaceae; Convolvuli pluricaulis herba]  *Cedrus deodara* (Roxb. ex D. Don) G. Don  [Pinaceae; Cedri deodarae lignum]  *Picrorhiza kurroa* Royle ex Benth. [Plantaginaceae; Picrorhizae rhizoma]  *Bacopa monnieri* (L.) Pennell  [Plantaginaceae; Bacopae monnieri herba]  *Nardostachys jatamansi* DC.  [Caprifoliaceae; Nardostachyos rhizoma et radix]  *Cyperus rotundus* Linn.  [Cyperaceae; Cyperi rotundi rhizoma]  *Cassia fistula* Linn.  [Fabaceae; Cassiae fistulae cortex]  *Terminalia chebula* Retz.  [Combretaceae; Terminaliae chebulae fructus]  *Vitis vinifera* Linn.  [Vitaceae; Vitis viniferae fructus] |
| 15. | *Raktadushtihar Yoga* | Anil A et al. (2015) | *Sariva*  *Musta*  *Katuka*  *Patha*  *Patola* | *Hemidesmus indicus* (L.) R. Br.  [Apocynaceae; Hemidesmi radicis]  *Cyperus rotundus* Linn.  [Cyperaceae; Cyperi rotundi rhizoma]  *Picrorhiza kurroa* Royle ex Benth. [Plantaginaceae; Picrorhizae rhizoma]  *Cyclea peltata* (Lam.) Hook. f. & Thomson [Menispermaceae; Cycleae peltatae radix]  *Trichosanthes cucumerina* Linn.  [Cucurbitaceae; Trichosanthis cucumerinae folium] |
| 16. | *Medhya Rasayana* | Dhananjay P et al. (2003) | *Brahmi*  *Mandukaparni*  *Shankhapushpi*  *Yashtimadhu*  *Guduchi*  *Jatamansi*  *Amalaki* | *Bacopa monnieri* (L.) Pennell  [Plantaginaceae; Bacopae monnieri herba]  *Centella asiatica* (L.) Urb.  [Apiaceae; Centellae asiaticae herba]  *Convolvulus pluricaulis* Choisy  [Convolvulaceae; Convolvuli pluricaulis herba]  *Glycyrrhiza glabra* Linn.  [Fabaceae; Glycyrrhizae glabrae radix]  *Tinospora cordifolia* (Willd.) Miers  [Menispermaceae; Tinosporae cordifoliae caulis]  *Nardostachys jatamansi* DC.  [Caprifoliaceae; Nardostachyos rhizoma et radix]  *Phyllanthus emblica* Linn.  [Phyllanthaceae; Emblicae officinalis fructus] |
| 17. | Cap. *Rakatchaphar* | Ruchika et al. (2011) | *Sarpagandha*  *Shankhapushpi*  *Jatamansi*  *Jahar Mohra Khatai Pishti*  *Moti Pishti*  *Ras Sindoor* | *Rauvolfia serpentina* (L.) Benth. ex Kurz  [Apocynaceae; Rauvolfiae serpentinae radix]  *Convolvulus pluricaulis* Choisy  [Convolvulaceae; Convolvuli pluricaulis herba]  *Nardostachys jatamansi* DC.  [Caprifoliaceae; Nardostachyos rhizoma et radix]  Serpentine stone compound  (CaCO₃, SiO₂, MgO – Jahar Mohra Khatai Pishti)  CaCO₃ from Pearl  HgS compound |
